# Profiguration, Active Ageing, and Creativity: Keys for Quality of Life and Overcoming Ageism

**DOI:** 10.3390/ijerph19031564

**Published:** 2022-01-29

**Authors:** Fidel Molina-Luque, Ieva Stončikaitė, Teresa Torres-González, Paquita Sanvicen-Torné

**Affiliations:** 1 Department of Sociology, GESEC-INDEST, University of Lleida, 25003 Lleida, Spain; teresa.torresgonzalez@udl.cat (T.T.-G.); paquita.sanvicen@udl.cat (P.S.-T.); 2 Department of English and Linguistics, DEDAL-LIT, University of Lleida, 25003 Lleida, Spain; ieva.stoncikaite@udl.cat

**Keywords:** profiguration, active ageing, intergenerational dialogue, socialization, wellbeing, creativity

## Abstract

This article is based on qualitative analysis of interviews and focus groups conducted with participants enrolled in the Senior Programme of the University of Lleida, the City Council of Lleida, and care homes, as well as professional workers in the field of gerontology and related areas. It presents the analysis of interviews focused on the participants’ life trajectories, ageing, creativity, self-perception, and quality of life. The study aimed to examine how creativity influences the maintenance and improvement of a sense of wellbeing in older adults, and to reflect on how the perception of old age and of oneself changes through creative activity and active engagement across the life span. The article is framed within a new concept in sociology and the social sciences—‘profiguration’, which is the key element in the promotion and strengthening of intergenerational interdependence, education, wellbeing, social participation, and active ageing.

## 1. Introduction

The COVID-19 pandemic transformed Europe and the world within the blink of an eye. The new ‘normality’, increased demographic ageing, and growing life expectancy are causing important changes in contemporary societies and will pose new challenges in the coming decades in different areas and disciplines including education, intergenerational coexistence, and social relationships. Therefore, there is an urgent need for better, innovative, and sustainable actions and solutions to adapt to changing realities and ensure that policies are fit for purpose in an era of major demographic changes. Although people live longer, many individuals are isolated from their family members, friends, and communities. Relatedly, a global decline in birth rates has provided fewer opportunities for children and young people to establish contact with individuals from different age groups; in particular, their grandparents. Loneliness and the lack of social interaction have become especially pronounced during the COVID-19 outbreak, which acted as a magnifying glass and further exposed increased physical and social distancing as well as age-related discrimination and hate speech against older adults [1,2]. 

Lockdowns, curfews, face masks, and sanitisation measures are policies that have been adopted by many countries to stop the current pandemic. Although nobody has been immune to these new regulations, the older segments of society have paid the greatest price, resulting in high death rates and increased marginalisation and loneliness of older adults [3,4,5]. Old-age loneliness and diminished in-person social encounters have dangerous consequences as they significantly contribute to physical and mental illnesses and poor quality of life [6]. Taking these issues into account and following one of the most exemplary initiatives of the 2020 European strategy to improve quality of life and social participation of older adults, there is a growing need to promote better intergenerational relationships, educational initiatives, and innovative actions, in order to contribute to the wellbeing of older adults and foster novel opportunities for their active ageing [7,8,9]. Profiguration and creativity are given particular attention in this study because, as will be shown, they can lead to more positive visions of age and ageing and foster intergenerational relationships, self-esteem, and the wellbeing of older citizens.

### 1.1. Profiguration

The methodological framework of this study is based on a new concept in sociology and the social sciences—‘profiguration’. The concept of profiguration (and ‘profigurative socialisation’) is a key element in improving coexistence and the relationships between individuals of different generations and age groups [10,11,12,13]. This neologism refers to the promotion and strengthening of intergenerational interdependence, education, social participation, and active ageing for older citizens. Profiguration is also closely linked to the need not only to advance active ageing but also to foster intergenerational dialogue, creative actions, and social innovation, and to eradicate both old and young ageism [14,15,16,17]. Profiguration is characterised by participatory, cooperative, integrated, and comprehensive education to promote a change that is manifested in collaborative decisions and dialogical, but not hierarchical, ways of interaction between various generations. Additionally, profigurative cultures and societies create interdependence between people at different life stages and help overcome loneliness, marginalisation, hate speech, and ageism. 

Profiguration also expands the types of socialisation and cultural transmission identified by the anthropologist Margaret Mead [18]. According to this scholar (1970), ‘prefigurative’ refers to the process of learning from children, while ‘postfigurative’ refers to the process of learning from older adults. Profiguration, wedded to co-operative intergenerational learning, transmission of knowledge, and research, enhances the importance of the interaction between research and teaching with a special focus on ‘prefigurative’ and, in particular, ‘postfigurative’ cultures. Since profiguration is connected to strengthening intergenerational relationships and coexistence between different age groups, it provides a new vision for mitigating and overcoming ageism and the generation gap that Mead (1970) described as planetary and universal. 

In the light of Mead’s defined ‘prefigurative’ and ‘postfigurative’ cultures of learning and transmission of knowledge, which she regarded as crucial for intergenerational dialogue and the creation of collective solidarity and collaboration, this study encourages actions in which older adults are given possibilities to engage in ‘profigurative’ socialisation and benefit from intergenerational interaction and dialogue [10]. Combining ‘prefigurative’ and ‘postfigurative’ cultures allows both older and young people to become active agents in decision-making processes, co-creation, and transmission of knowledge, which are much needed to foster intergenerational coexistence and a better understanding between different cohorts. Relatedly, profiguration draws on the sociologist Norbert Elias’ [19,20] concept of ‘figurational sociology’, which refers to a research tradition in which figurations of individuals—developing networks of interdependent humans—become the unit of research without separating an individual from society and, in turn, foster a form of continuum. According to Elias, as cultures and societies evolve and become more complex, interdependent, and dense, these processes, which he calls ‘the civilising process,’ cause changing behaviour patterns and social practices. Such actions create increasing demands on people to adjust their emotions and performance in ways that align with the expectations and requirements of the social networks to which they belong. 

### 1.2. Creativity

Along with profiguration, creativity has been given a special focus in this study. The relationship between creativity and intergenerational relations is closely wedded to the concept of profiguration and the idea of interdependence. Relatedly, creativity has an individual component and, at the same time, a social factor, because individualisation develops during the processes of socialisation [21]. Creativity is also linked to the transcendence of individual and social productions and is regarded as an inclusive articulation of the imaginary and reflective processes. It is related to historical and individual memory and reinforces individual, group, and community identities. According to the Royal Spanish Academy (Real Academia Española), Spain’s official royal institution for guaranteeing the stability of the Spanish language, creativity is defined as the power and ability to create or the facility to invent. Yet, one of the main challenges with creativity is that it is difficult to measure and to give a quantitative value because creativity is highly subjective and constantly evolving across the life span. It is also generally assumed that creative drives decrease in later life, in particular, around age sixty or thereabouts, while the greatest creative power is claimed to occur around one’s twenties or thirties [21]. 

However, traditional and stereotypical notions about creativity and later life do not contemplate the sociocultural and relational components or take into account the connections of lived experiences. In fact, the research literature shows that the meanings that older adults attach to their life cycle and, especially, to their experience of the final years, can reveal important aspects of the development of their creativity and self-perception [22,23,24,25,26]. Creative expressions can also provide a nuanced picture of older adults’ inner worlds and their ageing experiences from multiple perspectives and beyond empirical and medically oriented research. In fact, studies reveal that later life can be a period of high creativity that makes it possible to pursue ones’ goals and dreams in novel ways [8,21,24,27]. Late-life creativity (sometimes defined as ‘saging’) is dynamic and multi-layered, and can be expressed by “changing occupations, careers, and professions, or starting a new one; setting new goals or redirecting old ones; moving on to new ways of thinking and modes of imagination; posing new questions and pursuing unfamiliar lines of inquiry; searching within oneself for submerged talents and recharging hidden interests; and behaving in ways that were not just different but better than earlier efforts” [21] (p. vii). 

Creativity in later life stages is also a significant “source of self-discovery and self-creation” [26] (p. 3), as well as a way to achieve more meaningful living and a higher degree of self-confidence and maturity in older age [26,27,28,29]. Yet, although increased self-assurance and self-esteem are commonly associated with old age and have positive connotations, they are not natural consequences of living longer for they require effort and reflection, which is associated with the development of wisdom [30]. Seeking sense in our lives is basic to the human condition, creativity, inner strength, and knowledge, all of which become more imperative as people approach their final years [30]. Thus, the power of the creative drive can be seen as a tool that gives a sense of transcendence, wisdom, wellbeing, and self-fulfilment in advanced stages of the life course and offers new understandings about ageing and older age. This study challenges the assumption that creativity is related to youthful energy and reveals that the power of youthful creativity is a cultural construct that depends on dominant ideologies about age in different historical periods [31]. Additionally, the results of this research show that creative actions lead to increasing cognitive capacities, self-confidence, and more positive views of old age, and contribute to fostering intergenerational relationships. 

## 2. Materials, Objectives, and Methods

This study is based on qualitative analysis of interviews and focus groups conducted with the participants enrolled in the Senior Programme of the University of Lleida (henceforth referred to as UdL) in Catalonia (Spain). The same qualitative analysis was carried out with professionals working in the field of gerontology and related areas (nurses, occupational therapists, social workers, etc.), who were neither participants in the senior courses nor had a direct relation or contribution to the programme. The study also involved other older adults who were not participants in the UdL senior courses, but took part in local entities such as the City Council of Lleida and care homes that endorse creative activities, social participation, and active ageing. This selection allowed us to triangulate the information between the participating older adults and the professionals, since their opinions offered a valuable contribution and helped expand and contrast information, based on their professional experiences and knowledge. 

Triangulation offers different possibilities, among them data triangulation, researcher triangulation, theoretical triangulation, methodological triangulation, and multiple triangulation [32,33,34]. Its foundation is mainly focused on the idea that methods are instruments to investigate a problem and facilitate its understanding [32] (p. 75). In this study, we used data triangulation, researcher triangulation, and methodological triangulation between various methods. In particular, we employed different research techniques within the qualitative methodology based on in-depth interviews and focus groups, in order to expand the scope of the research and achieve the objectives. Data were collected in different places to check coincidences and they included different samples of subjects as well as several researchers in the field. The combination of different methods made it possible to use the strengths and mitigate the limitations or weaknesses of each method, cross-check data, contrast data, and observe whether the same conclusions were reached [32]. A joint analysis of the data also allowed biases in the research to be overcome and favoured a richer, more plural, and more complete analysis of results, in addition to giving the study a higher quality and coherence [32,33,34].

Triangulation occurred between the different profiles of the interviewed people: older adults from care homes, older adults (the term ‘senior students’ and its consistent use throughout the text refers to those older adults over 55 who are involved in the Senior Programme of the UdL) who do not reside in care homes but participate in university and municipal training courses, and professionals in the field of gerontology. The type of sampling was non-probabilistic—we chose an ‘intentional sampling’ method by selecting the participants based on their knowledge as researchers specialising in the subject matter, and collaborators in different fields of study. The participants were recruited using the so-called ‘snowball sampling’ method, also known as the chain-referral sampling method, which includes purposive sampling. We started by establishing specific profiles with a small sample of known individuals and then expanded it by asking those initial participants to identify others that could participate in the study. The determined profiles were chosen according to their age, gender, and participation in creative activities promoted by the UdL, the City Council of Lleida, and care homes. In summary, it was an intentional sample based on the profiles identified by the research team and the study objectives.

The interviews and focus groups took place in the classrooms of the UdL, the City Council of Lleida, and care homes. The main fieldwork was carried out by two specialised members of the research team. The participant observations were performed over two academic years in the subjects offered by the Open Classroom Unit (*Aula Oberta*) of the Senior Programme of the UdL. The interviews were recorded and transcribed (the average duration was between 35 and 45 min). For the complete analysis, the study involved four researchers from the team. The direct work of the first analysis was performed using the ATLAS.ti v8 programme and carried out by one of the four researchers who specialises in qualitative analysis with this software. The specialist used the coding tools for the subsequent interpretation of the data obtained by the group of involved researchers. The reliability, trustworthiness, and validity of this study were based on two rigorous methodological rules. The first was grounded in the validity of the experts who rigorously reviewed each of the main codes and its respective sub-codes to ensure that they were in line with the objectives of this investigation and followed the necessary content validity. On the other hand, the sociologists of the study ensured that in the areas of human behaviour and social sciences, and especially in the field of health, the validity of the instrument was linked to the understanding of the instrument [35] (p. 234), which applies to data collection methods that the participants interpret as responding to certain stimuli. In this qualitative study, the informants responded to questions and phrases related to the research topic. During the analysis of this study, the main codes and their respective sub-codes arose from the responses related to quality of life and a sense of wellbeing that older adults claimed to feel in relation to the creativity developed in this period of their lives. In summary, the stimulus applied during the interviews focused specifically on the topics related to lifelong learning, quality of life, and physical, subjective, and emotional wellbeing that, as the results reveal, continue into later years. The data obtained in this investigation verified that the participants clearly understood the questions, and therefore the understanding and the validity of the instrument were also checked. Likewise, in order to eliminate biases and confusion, different interviews were conducted both individually and in groups with people whose profiles were similar to the selected sample of the study. This was reflected in the homogeneity of the data obtained, which determined a coherent, organized, and relevant coding in accordance with the main objectives of the research.

The second methodological and also rigorous rule regarding the reliability, trustworthiness, and validity of this study was related to the triangulation process between the informants (older adults from the Senior Programme of the UdL, the City Council of Lleida, and care homes, as well as the professionals) and different qualitative research techniques (participant observation, interviews, and focus groups). The data obtained were continuously compared and analysed, building on empirical categories that allowed the results to be better explained. The findings were incorporated into the analysis process as an ongoing feedback and reassessment activity [35,36,37,38]. Their internal reliability was also oriented towards the level of interpretive agreement between different observers and evaluators, ten judges from the consolidated research group, and expert researchers who advised on the process, having previously obtained the necessary consensus. 

In total, eighteen interviews were conducted: eight with four female and four male professionals, and ten with five women and five men aged between 64 and 93. Additionally, three focus groups of six, eight, and six people (six men and fourteen women aged between 58 and 93) were carried out. The two main objectives of the study were:

1. To assess how creativity influences the maintenance and/or improvement of the quality of life and a sense of wellbeing in older adults who have started creative activities in later life, such as storytelling, creative reading, or writing workshops.
2. To reflect on how the perception of old age, ageing, and of oneself changes or does not change through creative activity in later life stages.

As the most appropriate methodology to achieve these objectives, we chose a qualitative methodology based on an intentional sampling of people who participated in activities that can help promote creativity. The interviews and focus groups with older adults allowed for better reflections on, and evaluations of, the influence of creativity on the quality of life and wellbeing of older adults. In addition, this methodology was contrasted with the experience of participant observation by researchers, and with interviews with professionals who have first-hand experience in such methods.

The Senior Programme of the UdL is a degree that is aimed at fostering and encouraging older adults’ lifelong learning, social participation, active ageing, and creativity, as well as intergenerational coexistence within the context of higher education. The four-year structure and the continuous assessment process comply with the general guidelines of the European Higher Education Area (EHEA) and the European Credit Transfer System (ECTS). The Senior Programme offers people over 55 numerous possibilities for broadening and deepening their previous knowledge by promoting educational learning, research, and service to society; improving social integration and participation; providing new opportunities to people from different professional backgrounds to extend their knowledge in other areas of study; encouraging personal and professional development, creative actions, tolerance, coexistence, and sociocultural, political, and economic participation; stimulating human relations and cooperation; providing scientific and cultural tools to better understand current society, science, and culture; and facilitating access to the use of ICT.

Additionally, the older students have an opportunity to carry out complementary activities that are recognised by three ECTS for each course, and they can choose extra activities and courses offered by the UdL and the City Council of Lleida. The senior students are also offered different group discussions and cultural and class activities that allow them to develop critical thinking skills and put into practice their capacities and knowledge to facilitate interpersonal and intergenerational relations, peer learning and research, and their personal and social wellbeing. Moreover, the last two years of the programme are especially significant as they provide the senior students with an opportunity to enrol in research-related subjects and areas and give them the possibility of collaborating with different university research groups through various workshops, events, and activities organised by the UdL that also involve diverse age groups.

There are specific courses, called the Open Classroom Unit (*Aula Oberta*), which are designed for both university students and older adults, and hence are inclusive and intergenerational. Although specific metrics have not yet been developed to assess the proportion of students, approximately one third of attendees are young people and two thirds are senior students. This uneven attendance is mainly due to the timetables in which the courses are taught. Future actions are aimed at rescheduling and improving the academic offer in order to make the university more intergenerational and age-friendly. One of the goals of this study is therefore to collect data and feedback to enable better conditions to be offered for younger and older students and to increase their motivation and interest in such courses. The previous interest and integration of younger students were manifested in their positive assessment and experiences, their willingness to enrol again, and their encouragement for other classmates to take part in the Senior Programme. The attitudes of the younger university students in relation to intergenerational contact were assessed through continuous evaluation, classroom practice, and peer work between both younger and older participants.

## 3. Results

The direct benefits of the Senior Programme of the UdL were observed in the interviews and focus groups that were carried out between 2019 and 2020. Using the ATLAS.ti v8 programme, they were coded and divided into fourteen main code groups, from which fifty-six sub-codes were derived, obtaining a total of seventy encodings. From those seventy encodings, the co-occurrences were qualitatively analysed, and fourteen conceptual networks were elaborated to identify and interpret the characteristics and factors related to creativity, a subjective sense of quality of life, learning processes, self-perception, and ageing (see Table 1). 

Before discussing the results, we explain the most important concepts and what they comprise in this study.

### 3.1. Active Ageing

The concept of active ageing aims to break down the often-negative image of the ageing process, which is socially and culturally linked to the idea of dependency, lack of productivity, frailty, and the notion of the end of life [31]. In this study, active ageing is understood as the involvement of everyone in social roles—older adults, citizens, and professionals—in the fields of politics, education, and public policy. It aims to make this stage of life more visible and to highlight older people’s rights and opportunities rather than their needs [39] (p. 27). Active ageing also implies observing and understanding later life not as a problem or burden but as an opportunity and a challenge in a holistic sense. According to the WHO, the ageing “of the population is one of humanity’s greatest triumphs and also one of our greatest challenges” [40] (p. 75). (The concept of active ageing is not new. The World Health Organization (WHO) has been defining it since 1980s based on the Vienna International Plan of Action on Ageing. The most cited definition of active ageing coined by WHO describes ageing as “the process of optimizing opportunities for health, participation and safety in order to improve the quality of life of ageing people” [40] (p. 79).)

### 3.2. Quality of Life

Some authors directly relate quality of life with the trinomial notion of family, health, and education as indicators and key elements of a better life [41]. Yet, so-called quality of life is not the same as happiness since it encompasses the three main meanings: quality of the environment in which we live, quality of action, and subjective enjoyment of life, which also involves a perception of happiness that is understood as a complete appreciation of life as a whole [42]. Precisely, there is empirical evidence that demonstrates a clear relationship between a sense of happiness and ‘actions’, specifically in relation to mental and physical health and wellbeing. In this study, the improvement of the quality of life is related to the promotion of health and wellbeing via educational intervention, which is strengthened by a series of essential factors such as social values (responsibility, solidarity, cooperation, commitment), the participation of the individuals in community activities, and their integration into positive group activities (sports, reading, art), as well as personal development (happiness, wellbeing, creativity) [43].

### 3.3. Self-Perception

Biography theory refers to the understanding of biographical actions and constructions of meaning through a narrative about a person’s life course [44,45,46]. A story that is written or explained by the person who is the main character in the events has many valuable elements that can make the story more unique in comparison to the account of a researcher or scientist. Although the investigative task is fundamental in order to better understand a personal life story and later life, the first-person account has specific elements that include freedom of speech, freedom of decision about what and how to explain, and freedom about what not to reveal [44,45,46]. These aspects have been given special consideration in this study. 

### 3.4. Ageism

Ageism is discrimination based on one’s age. The term was first coined by the gerontologist Robert Butler in 1969 [47] to identify prejudice and discrimination towards members of a certain age group, mainly older adults. According to Butler, unlike other prejudices such as racism or sexism, ageism affects everyone because we all grow older. Ageism, in short, is a set of discriminatory attitudes and behaviours that express prejudice and contempt towards advanced age. It might also end by justifying social inequality based on violence and the abuse of older adults. 

However, ageism not only affects older sectors of society but is also visible among young people. While old age is associated with uselessness, poverty (aporophobia), or ugliness (or the proximity of death) [31], youth is often related to breaking the rules, violence, disobedience, or selfishness (also linked to the idea of ‘carpe diem’). In order to challenge ageism, the UN advocates the promotion of intergenerational solidarity through social programs, regardless of the area in which they are developed, especially taking into account the fact that ageing is understood as a worldwide process that must be improved. It is also crucial to take into account the fact that violence and discrimination against older people is caused by a lack of action, help, and collaboration, especially with regard to physical, emotional, and psychological abuse, social exclusion, loneliness, and contempt. These attitudes and behaviours must be carefully uprooted in areas ranging from public administration and education to the community (friends, family, and neighbours). Relatedly, ageist patterns must be tackled within a profigurative socialisation framework, which can and must be implemented in the educational system, social education, and even informal education through social networks, ICT, television, and leisure activities, in order to raise awareness about the existence of ageism and lack of respect towards people at any stage of their lives. This awareness also includes the new vision of equity and solidarity that goes beyond the mercantilist vision of the ‘economic’ value (production and consumption) of people [13].

### 3.5. Obtained Results

The quotations taken from the interviews and focus groups are arranged by the main themes/codes, which very often intersect and blur into each other. The main codes, which refer to the positive relationships between socialisation and creativity, health and wellbeing, and satisfaction in learning, as well as an increased sense of self-confidence and personal growth, are visible in almost all the older adults’ responses. For instance, a female student, aged 68, enrolled in the Senior Programme of the UdL, highlighted that lifelong learning and expanding her previous knowledge and stimulating her creativity had helped her improve her health and sense of wellbeing and happiness. To her, “creating is good for your health, because you are happy, it makes you happy”. The same senior participant also stated that it was a high time she started engaging with creative expressions and acquiring new knowledge because, in later life, time becomes the enemy and reduces the possibly of learning and growing personally: “I don’t know how much time I have left… and I have to do a lot of things.”

Similar thoughts are expressed by another female student, aged 62, who stated that she had felt more invigorated and active, and had even “become younger”. Like the previous participant, she admitted that being involved in the Senior Programme “has given [her] vitality” and made her “so happy” and fulfilled. Emotional wellbeing, a proactive attitude to life, and interest in lifelong learning are also expressed in her novel discovery of herself in terms of increased capacity for concentration and the ability to work better: “I am a very disciplined person in everything in general; […] I really like studying”. She has also admitted that:

I have realised that despite [my old age], you see that you are alive, that you have a great mind, that you have a great memory, a capacity, that you can study many subjects at the same time—I was personally very surprised by this, and I feel fabulous now (laugh).

According to this participant, being part of a broader university community has also helped her to feel more fulfilled and satisfied with life and herself:

I value myself a lot more now because I see that I really have many abilities and I had discovered some new skills; I’m alive and […] still worth a lot, you’re worth a lot and you have a lot to offer to others and to yourself too.

Better adaptation to retirement, active ageing, increased socialisation, creativity during ageing, and satisfaction with life are also visible in the arguments of a male senior student, aged 66. According to him, he is happy to be able to “meet new people and learn a lot, which has an added value” to his retirement stage and life in general:

That is to say, what kills people is the boredom. The routine that gets nowhere, I think there are enough things to do to feel important. […] you can feel useful even if you don’t do anything, it doesn’t mean that you don’t do things at home, collaborate… But you feel good about yourself.

The positive relationship between creativity and mental wellbeing and the importance of lived experiences for later-life creativity are also reflected in his belief that creativity is the cause of mental lucidity. According to the participant, “the more lucid one is, the more quality of life one has”. Another male participant in the activities organised by the City Council, aged 76, also emphasised the positive position versus lived time/time to live, active engagement with life, mental wellbeing, and better quality of life:

Three years ago, I started a course with the teacher […] and it lasted for three years. The truth is that I’m fine, apart from being able to express my feelings, it helps me to think, which I think is good for ‘conserving neurons’. It encourages me to see that when I expose my poetry, or my writings, they have a good reception, and this helps me to continue; I feel that maybe I should publish, not in order to sell anything, but at least to make it known to other people and express the ideas that flow in my brain, in my way of thinking.

Being part of the community has helped him to become more social, engaged, and self-confident: “meeting other students, listening to what they present, and what I present to them, has helped me a lot. I am excited. I think I’m fine physically and mentally.” The participant has also started to feel more proactive and challenged age-related stereotypes about passivity and inactivity in later years. He has felt more “useful, because when you reach a certain age, it seems that you are already like an old piece of furniture, that you can’t think or write. It’s a challenge.” The idea that creative actions, social participation, and being engaged contribute to feeling ‘younger’ and invigorated is also expressed by a female senior student, aged 68, who has noted that: “creativity makes you younger because you are doing new things, and it is good for your health, because you are happy”. The positive relationship between creative activities and mental health is expressed by a male participant, aged 65, who has argued that being creative and active stimulates one’s “brain and neurons”. According to him, “there is less Alzheimer’s among people who are dedicated to the study, culture, music,“ thus, being mentally active ”is the basis that works for the rest of the body” and mind. Relatedly, another male student, aged 60, has argued that “every creative act generates satisfaction and, at the same time, self-esteem”. The same views are expressed by another female senior student, aged 60, who has compared the possibility of being part of the university to opening new doors to her life: “I have opened […] a lot of doors, even intellectually and at a creative level, because [creativity] opens your mind much more.” Her comments affirm the close relationship between creativity and better life quality, emotional wellbeing, self-esteem, and personal motivation. 

Being enrolled in the Senior Programme of the UdL has also given older adults an opportunity to become more familiar with the use of ICT, which helps them to be more engaged and connected. The same female senior student, aged 60, expressed positive views about the use of digital technologies by stating that “we do some PowerPoints and presentations… That’s fabulous! This kind of learning encourages people, because you feel that life gets longer when you are active and not passive.” Another female senior student, aged 71, has also pointed to the benefits of acquiring new digital skills: “I really like the use of ICT and I currently have three web pages, I like it a lot, this stimulates me.” Eagerness to learn and be engaged is also reflected in the comments of a male student, aged 60, in which the participant argues that the use of ICT is crucial to his personal development and creativity:

Technology is useful and it depends on the creative drive, obviously. […]. I mean, technology is here to stay, and it is very necessary, I think we cannot go against technology, we cannot be like those who said, ‘burn the machines, burn the industrial revolution!’

Willingness to learn more about digital technologies and devices is also expressed by another male student, aged 65: “I do not think that technology is bad, it’s much better to write using the Word document than using the graphic style pen.” Although the participant is interested in ICT, he is also critical about communication technologies and applications such as WhatsApp or Facebook, which, according to him, “can take away many hours from developing your creativity”. Another male senior student, aged 65, stated that he was grateful to the Senior Programme for giving him a chance to study many different subjects, including the use of ICT, which he did not have before: “at age 16 I already had to go to work, then my desire to learn and study were truncated […]. However, since I am at university now, I have this feeling that a door has opened in my brain.” 

The participants’ responses are corroborated by professionals involved in the study. A male occupational therapist, aged 45, regarded creativity as a means of being active, capable, and engaged. According to him, creativity leads to better quality of life: “when a person is creative it means that he is active. Therefore, he is already employing different functions of his body that will give him quality of life.” Relatedly, one of the professional female nurses, aged 46, pointed out that it was crucial to keep on learning and progressing across the life span in order to foster active living and the mental health of older citizens. According to her, “creativity helps you to improve facets… you learn… you develop new skills, it is related to pleasure, to the happiness of doing something you like”, and she emphasised that enrolling in the Senior Programme and creative actions enhances people’s quality of life and self-assurance. The professional female nurse noted that society does not provide individuals with enough chances to be creative, and in some cases even denies those opportunities; however, enrolment in the Senior Programme of the UdL offers the possibility to older adults of fostering their creative capacities and, at the same time, challenges the traditional notion that creativity is related to youthful energy. To her, old age is a not a stage of frailty and inactivity but rather a stage of becoming and personal growth: “I mean, that sometimes society or people think that older adults have no ability to learn new things and internalise them.” She has also observed that in earlier life stages many people did not have many opportunities to study because of work and family obligations (“I think adult life is full of obligations, working… creating a family”). The possibilities of engaging in creative activities and broadening their knowledge and interests were limited. However, the Senior Programme of the UdL was a new opportunity for many older adults. Relatedly, another interviewed female social worker, aged 47, stated that the possibility of learning and broadening one’s horizons was a means of therapy, social integration, active ageing, and meaningful participation in society: “I say that if you do one thing and enjoy it, it is double, it is 100% therapy, whatever it is.”

## 4. Discussion

As expressed by the participants of the Senior Programme of the UdL, creative actions help improve cognitive functioning and a sense of wellbeing in older age, and the results reveal that active engagement and social participation foster a feeling of usefulness and worth in later years. At the same time, the results of the study show that creative drives encourage interpreting and reinterpreting one’s life course and lived experiences, leading to higher self-esteem and self-confidence. According to the participants in the Senior Programme of the UdL, an improved sense of self, health, and mental wellbeing are also closely wedded to a feeling of happiness, pleasure, and vitality in older age. Additionally, the students in the programme have pointed to overcoming loneliness and the fears of starting new activities, such as taking part in different workshops or creative expressions. Participating in novel activities, enrolling in courses, and satisfactorily concluding them lead to better quality of life, expansion of previous knowledge, and an acquisition of a series of new ‘obligations’ (non-imperative) that generate socialisation habits and create a sense of usefulness in later life. The participants in the study also expressed that ageing is a not a fixed stage in life but rather an ongoing process of learning and of becoming. 

The study’s findings also show that use of ICT (also e-books, video calls, and computer programmes such as Word, PowerPoint, and others) helps older adults to stay engaged and connected, which fosters intergenerational dialogue and being updated about current societal issues. The acquisition of digital competence among older adults is crucial in supporting active ageing and lifelong learning, creating benefits across different generations, and narrowing the gap of the digital divide. Moreover, the use of ICT and learning throughout all life stages is one of the European Union programmes aimed at promoting active ageing and strengthening the contribution of older citizens to the learning of younger generations, which stimulates better intergenerational bonding, raises the awareness of how older adults can contribute to society, and closes the distance between the younger and the older generations. The Senior Programme of the UdL and related intergenerational and creative activities help older adults to actively engage in life, realise their potential as full citizens, and make an active contribution through participation in their local communities, voluntary work, and intergenerational courses. Such activities also create a more positive public image of later life that is not portrayed through the lens of the narrative of decline, according to which older adults are perceived as frail, dependent, and passive [31]. On the contrary, the participants show that older age can be an enriching period in life that should not be feared but rather embraced with positivity and seen as a stage that offers new possibilities to learn and grow both personally and professionally, and, eventually, to get closer to their inner selves. Moreover, intergenerational activities, the use of ICT, and shared courses not only bridge the gap between different generations but also encourage meaningful intergenerational learning and dialogue, activism, and social participation within university–community partnerships [7]. 

It is also important to bear in mind that senior students are not mere objects of study but active agents and participants in decision-making processes and in research and teaching methodologies. Being an integral part of university–community settings can foster critical thinking, problem-solving skills, cognitive capacity, and active social engagement, all of which contribute to active ageing and wellbeing in later years of life [8]. Although it is difficult to measure creativity and social and civic engagement in a strictly quantitative way, both formal and informal conversations and feedback from the participants show more willingness to be active and participatory and reveal an increased involvement in social life, thus helping to combat loneliness. Moreover, taking into account the fact that the city of Lleida is relatively small, information about the students’ opinions and experiences is shared more easily and faster, both inside and outside the university setting (in the City Council, neighbours’ associations, cultural gatherings, social events, senior clubs, etc.). 

Ultimately, the results of the study reveal that the participants could merge both ‘prefigurative’ and ‘postfigurative’ cultures as previously identified by Mead [18] by helping younger generations in their process of growth, while in turn older adults could acquire new skills and knowledge from younger people. Profiguration was especially visible in interviews and focus groups when discussing the complexities of growing older, sometimes comparing them to the transition from childhood to youth and adulthood. The senior students critically reflected on their processes of maturation, personal growth, ageing, and lived experiences from a life course perspective. For instance, a specific action was carried out among children and older adults in one of the care homes in Balaguer (a city near Lleida, Spain), where some schoolchildren sent a letter to older adults and eventually visited them. This encounter resulted in shared life stories, mutual learning, and intergenerational dialogues. Another example of profiguration is a course on Mediation and Conflict Resolution in the Open Classroom Unit (*Aula Oberta*), organised by one of the authors of this article, in which ageism, age-based marginalisation, intergenerational relationships, and creativity were discussed with different age groups. The course was aimed at orienting the professionals on how to better implement creativity and intergenerational aspects, foster profiguration, value the course methods and structure, and inform and disseminate the research study for future actions. More intergenerational courses could teach students how to manage conflicts, how to transform them into opportunities, and how to solve them in sustainable and creative ways. Such actions could become a site and an example of profiguration in the university environment for both young and older students, where intergenerational actions enrich personal relationships and provide opportunities to share previous knowledge and skills by placing a special emphasis on interdependence, creativity, and lifelong learning.

One of the limitations of this study is that a credit-bearing university course may not be appropriate for, or benefit, all older adults, who come from different backgrounds and life paths. However, the objective of the study was to find out how creativity in later life can be strengthened with specific activities that are not only part of the university setting and programmes but also include different social and creative actions offered by other entities. 

## 5. Conclusions

This study examined how creativity influences the maintenance and improvement of a subjective sense of quality of life and wellbeing in older adults who have started some creative activities in later years. It also evaluated how an individual perception of old age and ageing, and of oneself, changes through creative activity in later life stages. The findings, based on first-person accounts of the participants, show that engaging in creative actions can positively influence the process of growing older and helps foster a feeling of self-satisfaction and empowerment. As explained by the participants in the interviews, personal narratives, and focus groups, attending and actively participating in training courses and creative actions such as writing makes them feel that they have enhanced their self-esteem and quality of life. They perceive it and, thus, live it that way. The subjective perception of increased wellbeing, as they affirm, is transformed into an objectified reality of a physical and mental state that makes them feel much better about themselves and the process of growing older. It also leads them to encourage acquaintances, friends, and relatives to get involved in activities of this kind. These aspects are important as they also show how creative actions can allow older adults to become more involved, create, share, and improve their sense of wellbeing, which translates into personal improvement in a holistic sense. Being part of a dynamic university community that brings different age groups together and actively engaging in social and civic activities provides older adults with novel opportunities to broaden their knowledge and skills, and feel more energetic and self-confident. 

At the same time, many older adults have pointed to the opportunity to become more familiar with the use of ICT and digital devices, which make it possible to be more engaged, updated, and connected, and bridge the gap between different age groups. The participants’ responses also demonstrate that older age can be an enriching and invigorating life stage that provides older adults with novel opportunities and critical thinking skills, and fosters a sense of happiness and self-worth, and intergenerational connections. The findings show the relevance of developing the profiguration theory, which refers to the reciprocal and active social contract between generations through concrete actions with creative activities such as narrative or writing. The results also affirm that such actions help create more positive images of old age and ageing, and challenge discriminatory age stereotypes.

In order to provide older adults with positive experiences and learning opportunities, it is imperative to undertake and promote intergenerational actions and creative expressions that appeal to different age groups and enhance their creativity and interest in lifelong learning. Collaborative rather than hierarchical socialisation and active participation based on supportive intergenerational relationships, in which educators combine their roles as researchers and teachers, can further enhance profiguration and provide people with novel learning opportunities, fostering social inclusion and active ageing [10,11,12,13]. However, there is a necessity not only to advance active ageing policies but also to favour intergenerational relationships in a profigurative society through intergenerational dialogue, which would help overcome both young and old ageism and bridge the gap between different cohorts. To ensure good functioning and a continuation of intergenerational activities and programmes, there is a need to involve educators that have been trained in intergenerational issues and to provide senior students with creative and engaging opportunities to encourage them to take an active part in both academic and non-academic settings and local communities [8]. 

However, very importantly, intergenerational education and coexistence should be approached in complex ways, taking into account their content and meaning [48]. The ‘profigurative socialisation’ should materialise in the daily practice of an intergenerational social contract in which young people (in this study, university students) and older adults are given opportunities for intergenerational exchange and the sharing of knowledge in innovative and creative ways by placing a special emphasis on interdependence, creativity, and lifelong learning. These issues should become priority aspects in educational agendas for all ages and an important part of many global processes, including family relationships, healthcare, leisure time, economics, and public administration and policies. 

Ultimately, the findings of this study significantly contribute to the scientific community and research, as they show the relevance of the concept of profiguration and its consolidation as a new theoretical element that adds to the importance of intergenerational relations and to improving a sense of wellbeing of older people. It also contributes to rewriting the narrative of decline and combating negative notions of growing older. Relatedly, this research demonstrates that creativity does not diminish with age, because it is a lifelong learning process of becoming and personal growth that can be further enhanced by creative activities and training programmes for older adults, to better consolidate their ageing. 

## Figures and Tables

**Table 1:** Codification of results.

Main Codes	Sub-Codes	Co-Occurrences
1. Socialisation/creativity (37-3)	1.1 Meeting new people	12-1
	1.2 Sharing and having fun	27-1
2. Creativity/health and wellbeing (192-9)	2.1 Mental wellbeing	60-1
	2.2 Physical wellbeing	36-1
	2.3 Emotional wellbeing	112-1
	2.4 Proactive attitude	95-1
	2.5 Personal growth	10-1
3. Creativity/self-confidence (112-5)	3.1 Creativity/self-esteem	54-3
	3.2 Creativity/self-image	4-1
4. Motivation for creativity (89-6)	4.1 Internal and/or personal motivation	72-2
	4.2 External motivation	38-1
5. Satisfaction in learning (83-5)	5.1 Opportunity to learn	19-2
	5.2 Learning as a positive experience	49-2
	5.3 Learning about ICT	11-1
6. Creativity during ageing (222-8)	6.1 Passive creativity	13-1
	6.2 Active creativity	155-1
	6.3 Creativity/quality of life in older age	97-2
	6.4 Whether there is a reading/writing relationship	22-1
	6.5 There is no reading/writing relationship	10-1
7. Passive retirement (22-1)		22-1
8. Memories and lived experiences (45-1)	8.1 Nostalgia	2-1
	8.2. Positive memories of the past	24-1
	8.3 Rejection of memories of the past	5-1
	8.4 Refusal to talk about hard subjects: death, love, etc.	5-1
9. Lived experiences and their influence on creativity (102-8)	9.1 Creativity in childhood/adolescence	17-1
	9.2 Creativity developed in adulthood/older age	73-1
	9.3 Accumulated wisdom	60-1
	9.4 Emotions and feelings as a creative theme:	30-1
	9.4.1 The family as a creative theme	5-1
	9.4.2 First love as a creative theme	2-1
	9.5 Everyday life as a creative theme	7-1
12. Position versus lived time/time to live (119-9)	12.1 Life satisfaction	69-2
	12.2 Sadness, anger, apathy, or disappointment with life	12-1
	12.3 Time is running out for living	9-1
	12.4 Time arrangement	37-1
	12.5 Past experiences constantly emerge	14-1
	12.6 I am still healthy/I feel useful	11-1
	12.7 Seeking new perspectives and roles in life	10-1
13. Active retirement (197-8)	13.1 Retirement, creativity, and learning	53-1
	13.2 Retirement and various activities	81-2
	13.3 Retirement and socialisation	55-2
14. Factors that encourage creativity (38-4)	14.1 Remoteness from complexes and/or fears	5-1
	14.2 Personal incentives and/or motivations	23-1
	14.3 Socialisation	10-1
	14.4 Economic factors	2-1

## Data Availability

The data presented in this study are available at http://www.envejecimientoycreatividad.udl.cat/es/linea2/, accessed on 21 January 2022.

## References

[1] Ayalon L., Chasteen A., Diehl M., Levy B.R., Neupert S.D., Rothermund K., Tesch-Römer C., Wahl H.W. (2020). Aging in Times of the COVID-19 Pandemic: Avoiding Ageism and Fostering Intergenerational Solidarity. J. Gerontol. Ser. B.

[2] Gullette M.M., Barry E., Vibe Skagen M. (2020). Afterword: When Age Studies and Literary-Cultural Studies Converge: Reading ‘The Figure of the Old Person’ in An Era of Ageism. Literature and Ageing.

[3] Higgs P., Gilleard C. (2021). Fourth Ageism: Real and Imaginary Old Age. Societies.

[4] Fallon A., Dukelow T., Kennelly S.P., O’Neill D. (2020). COVID-19 in Nursing Homes. QJM Int. J. Med..

[5] Heidinger T., Richter L. (2020). The Effect of COVID-19 on Loneliness in the Elderly: An Empirical Comparison of Pre- and Peri-Pandemic Loneliness in Community-Dwelling Elderly. Front. Psychol..

[6] Cohen-Mansfield J., Hazan H., Lerman Y., Shalom V. (2016). Correlates and Predictors of Loneliness in Older-Adults: A Review of Quantitative Results Informed By Qualitative Insights. Int. Psychogeriatr..

[7] Agmon M., Doron I., Ergon-Karlin S. (2018). Gerontological Activism: An Example of an Intergenerational Academic Course within a University–Community Partnership. Educ. Gerontol..

[8] Molina-Luque F., Casado N., Stončikaitė I. (2018). University Stakeholders, Intergenerational Relationships and Lifelong Learning: A European Case Study. Educ. Gerontol..

[9] García Lizana F. (2013). European Innovation Partnership on Active and Healthy Aging: Moving from Policy to Action. Gac. Sanit..

[10] Molina-Luque F., Molina-Luque F., Gea-Sánchez M. (2017). Calidad De Vida y Socialización Profigurativa: Consideraciones Éticas Sobre Investigación En Educación y En Salud. Educación, Salud y Calidad de Vida: Nuevas Perspectivas Interdisciplinares e Interculturales.

[11] Molina-Luque F. (2019). Profiguración, Acción Creativa Intercultural e Innovación Social: Renovarse o Morir en Rapa Nui (Isla de Pascua/Easter Island). Rev. Latinoam. Estud. Sobre Cuerpos Emoc. Soc. (RELACES).

[12] Molina-Luque F. (2020). The Art of Living as a Community: Profiguration, Sustainability, and Social Development in Rapa Nui. Sustainability.

[13] Molina-Luque F. (2021). El Nuevo Contrato Social Entre Generaciones. Elogio de la Profiguración.

[14] Bratt C., Abrams D., Swift H.J. (2020). Supporting the Old but Neglecting the Young? The Two Faces of Ageism. Dev. Psychol..

[15] Bratt C., Abrams D., Swift H.J., Vauclair C.-M., Marques S. (2018). Perceived Age Discrimination across Age in Europe: From an Ageing Society to a Society for All Ages. Dev. Psychol..

[16] Butler R.N. (2005). Ageism: Looking Back over My Shoulder. Generations.

[17] Duncan C., Loretto W. (2004). Never the Right Age? Gender and Age-Based Discrimination in Employment. Gend. Work. Organ..

[18] Mead M. (1970). Culture and Commitment: A Study of the Generation Gap.

[19] Elias N. (1990). La Sociedad de los Individuos: Ensayos.

[20] Elias N. (2010). Sociología Fundamental.

[21] Lindauer M.S. (2013). Aging, Creativity, and Art: A Positive Perspective on Late-Life Development.

[22] Cohen-Shalev A. (1989). Developmental Changes in Creative Production from a Life-Span Perspective. J. Aging Stud..

[23] Cohen-Shalev A. (2008). Sailing on Seas of Uncertainties: Late Style and Puccini’s Struggle for Self-Renewal. Int. J. Ageing Later Life.

[24] Casado-Gual N., Domínguez-Rué E., Worsfold B. (2016). Literary Creativity and the Older Woman Writer: A Collection of Critical Essays.

[25] Stončikaitė I. (2019). Ageing, Creativity, and Memory: The Evolution of Erica Jong’s Literary Career. Life Writ..

[26] Wyatt-Brown M.A., Wyatt-Brown M.A., Rossen M.A. (1993). Introduction: Aging, Gender, and Creativity. Aging and Gender in Literature: Studies in Creativity.

[27] Cohen G.D., Cole T.R., Ray R., Kastenbaum R. (2010). Creativity and Aging: Psychological Growth, Health, and Wellbeing. A Guide to Humanistic Studies in Aging: What Does It Mean to Grow Old.

[28] Randall L.W. (2013). Aging, Irony, and Wisdom: On the Narrative Psychology of Later Life. Theory Phycol..

[29] Randall L.W., Kenyon G.M. (2004). Time, Story, and Wisdom: Emerging Themes in Narrative Gerontology. Can. J. Aging.

[30] Baars J. (2012). Aging and the Art of Living.

[31] Gullette M.M. (2004). Aged by Culture.

[32] Aguilar Gavira S., Barroso Osuna J. (2015). La Triangulación De Datos Como Estrategia En Investigación Educativa. Píxel-Bit Rev. Medios Educ..

[33] Feria Avila H., Matilla González M., Mantecón S. (2019). La Triangulación Metodológica Como Método De La Investigación Científica. Apuntes Para Una Conceptualización La Triangulación Metodológica Como Método De Investigación. Didasc@Lia Didáctica Educ..

[34] Okuda Benavides M., Gómez-Restrepo C. (2005). Métodos en investigación cualitativa: Triangulación. Rev. Colomb. Psiquiatr..

[35] Sandín M.P. (2000). Criterios de Validez en la Investigación Cualitativa: De la Objetividad a la Solidaridad. Rev. Investig. Educ..

[36] Hernández-Sampieri R., Mendoza Torres C.P., McGraw Hill Interamericana (2008). Metodología de la Investigación: Las Rutas Cuantitativa, Cualitativa y Mixta.

[37] Martínez Miguélez M. (2006). Validez y Confiabilidad en la Metodología Cualitativa. Paradígma.

[38] Vivar C.G., McQueen A., Whyte D.A., Canga Armayor N. (2013). Primeros Pasos en la Investigación Cualitativa: Desarrollo de Una Propuesta de Investigación. Index Enfermería.

[39] Giró Miranda J. (2006). Envejecimiento Activo, Envejecimiento en Positivo.

[40] WHO (2002). Active Ageing: A Policy Framework.

[41] OECD (2013). Citizen Wellbeing Index.

[42] Veenhoven R., De Girolamo G., Becchi A., Coppa D.S., De Leo D., Neri G., Rucci P., Scocco P. (2001). Calidad de Vida y Felicidad: No es Exactamente lo Mismo. Qualita’ Della Vita e Felicita’.

[43] De Vincezi A., Tudesco F. (2009). La Educación Como Proceso De Mejoramiento de La Calidad de Vida de Los Individuos y de La Comunidad. Rev. Iberoam. Educ..

[44] Vilaseñor I. (2015). La Narrativa Del Paciente Como Herramienta Terapeutica. Pan Am. Fam. Med. Clin..

[45] Hernández F.J., Villar A. (2015). Educación y Biografías. Perspectivas Pedagógicas y Sociológicas Actuales.

[46] Dausin B., Hernandez F., Villar A. (2015). Aprendizaje Biográfico y Biograficidad. Reflexiones Para Una Idea y Una Práctica Pedagógicas en La Formación de Persones Adultas. Educación y Biografías. Perspectivas Pedagógicas y Sociológicas Actuales.

[47] Butler R. (2008). The Longevity Revolution: The Benefits and Challenges of Living a Long Life.

[48] Morin E. (2000). La Mente Bien Ordenada.

